# Extreme and Topological Dissipative Solitons with Structured Matter and Structured Light

**DOI:** 10.3390/nano9060826

**Published:** 2019-05-31

**Authors:** Nikolay N. Rosanov, Sergey V. Fedorov, Leonid A. Nesterov, Nikolay A. Veretenov

**Affiliations:** 1 Vavilov State Optical Institute, Kadetskaya Liniya V.O. 5/2, St.-Petersburg 199053, Russia; torrek@gmail.com; 2 Ioffe Institute, St.-Petersburg 194021, Russia; nesterovla@mail.ru; 3 Department of photonics and optical informatics, ITMO University, St.-Petersburg 197101, Russia; sfedorov2006@bk.ru

**Keywords:** dissipative optical solitons, switching waves, molecular aggregates, lasers with saturable absorber, topological solitons

## Abstract

Structuring of matter with nanoobjects allows one to generate soliton-like light bundles with extreme characteristics—temporal duration and spatial dimensions. On the other hand, structuring of light gives the possibility to form light bundles with complicated internal structure; their topology could be used for information coding similar to that in self-replicating RNA molecules carrying genetic code. Here we review the both variants of structuring. In the first variant, we consider a linear molecular chain and organic film interacting resonantly with laser radiation. Demonstrated are optical bistability, switching waves, and dissipative solitons, whose sizes for molecular J-aggregates can reach the nanometer range. We also discuss some theoretical approaches to take into account multi-particle interaction and correlations between molecules. In the second variant, light structuring in large-size laser medium with saturable amplification and absorption is achieved by preparation of the initial field distribution with a number of closed and unclosed vortex lines where the field vanishes. Various types of topological solitons, parameter domains of their stability, and transformation of the solitons with slow variation of the scheme parameters are presented.

## 1. Introduction

Reducing the spatial dimensions of optical (laser) radiation bunches is necessary for obtaining an extremely high concentration of radiation energy, for high-performance optical recording and transmission of information, and for a number of other applications. However, with the propagation of beams and light pulses as their sizes decrease, their spreading increases due to diffraction and dispersion effects, which reduces the efficiency of many optical applications. The possibility of focusing the radiation and, accordingly, modifying materials and recording information are limited usually to the order of the optical wavelength.

Plasmonic methods [1] in structures containing metals make it possible to obtain subwavelength structures of light, but they introduce significant losses due to absorption of radiation in the optical region of the spectrum. Compensation of laser radiation diffusion is possible in nonlinear optical media, which improves the transmission efficiency of information in fiber lines in the mode of solitons—radiation pulses that do not change their shape when propagate in a fiber with a nonlinear refractive index [2]. However, the formation of stable solitons in a bulk medium, which is necessary, for example, for transferring of 2D- and 3D-arrays of information, causes difficulties. These circumstances indicate the need to find new approaches to the ultimate compression of light structures.

This article provides an overview of research in two relevant areas, in which the use of resonant optical nonlinearity of media is common. In the first part of the review, the problem of obtaining soliton-like structures of light in nanostructures—high-molecular systems and organic materials—is considered. The promise of the latter was relatively recently demonstrated in articles [3,4]. The second part deals with the possibility of structuring not the material, but the light radiation, in the limiting mode of three-dimensional (volume) solitons with a controlled internal structure; this would allow one to encode and transmit information without distortion. Let us remind that the first part of the paper deals with nanomaterials like molecular J-aggregates and organic films, and the second part is devoted to laser solitons with complicated internal structures. Such solitons can be more efficiently formed and processed with nanostructured materials including quantum dots due to large dipole momenta of their electronic transitions. It is also possible that some of the structuring methods developed for radiation could be transferred to nanomaterials for their finer structuring.

## 2. Structuring of a Medium

An important problem is to understand what the ultimate degree of structures localization is, and how to reach it. In the literature, nanosized structures were reported for spin waves in antiferromagnetic lattices [5] and in magnetic contacts [6]; however, in the later approach, only one unit (information bit) could by recorded over the whole contact. It seems that the narrowest localized structures promising for a number of applications including molecular computing, can be formed on the nanosize, molecular level. The investigations of localized excitations in molecular, or even atomic chains were initiated by the classical works [7,8,9]. However, these and most of the subsequent publications were devoted to idealized, conservative systems without dissipation. Below we review the research of localized structures in dissipative molecular schemes with balanced energy input and output that support much more stable structures—switching waves (kinks, fronts) and dissipative solitons.

### 2.1. Molecular J-Aggregates

Oriented J-aggregates [10] of cyanine dyes can be attributed to the number of nanostructured materials with a molecular level of nano-objects. We will consider J-aggregates of cyanine dyes ${\left(\mathrm{PIC}:\mathrm{Cl}\right)}_{N}$ consisting of *N* molecules of pseudo-isocyanine chloride (PIC:Cl) forming a chain; the value *N* can reach hundreds and thousands. They have a collective (excitonic) mechanism of their resonant excitation in the optical region of the spectrum. This leads to strong, compared with the response of the components of the aggregate molecules, optical nonlinearities with subpicosecond relaxation times, which makes such nanostructures promising for a variety of optical technologies [10,11]. Aggregated cyanines form ordered supramolecular structures, like fibers with the width less than 3 nm, with the potential to transport efficiently energy over long distances [12]. This underlines the relevance of development of a more complete theory of collective nonlinear optical response of J-aggregates and a wider analysis of their potential applications. 

Of considerable interest is the prospect of using J-aggregates in schemes of molecular data storage. The bistability necessary for long-term data storage in single J-aggregates was predicted and studied in References [13,14], and in an ensemble of aggregates (in a thin film) in References [15,16,17]. The theory in these papers was based on the approximation of a one-particle density matrix without taking into account three-particle and higher order interactions, as well as correlations between molecules.

Next, we present the further development of these studies in two directions. First, we overview the development of a more complete theory taking into account three-particle interactions and correlations between molecules, as well as the application of this theory to the analysis of bistability and (modulation) instability in J-aggregates [18,19,20,21,22]. Second, we present more brief information about the “nanosolitons” predicted in Reference [18] and investigated in References [19,20,21]: discrete dissipative solitons in J-aggregates of molecular (nanometer) sizes.

#### 2.1.1. Model of J-Aggregate and Governing Equations

An individual J-aggregate is modeled by a chain consisting of three-level molecules, the first and second levels of which are associated with similar levels of other molecules through dipole-dipole interaction; the interaction with the third level is carried out using the mechanism of exciton-exciton annihilation. In this case, the external monochromatic supporting radiation with linear polarization can directly interact only with the transition 1→2. It is also assumed that the transition frequency 2→3 is close to the transition frequency 1→2. In the absence of radiation transitions from the first and second levels to the third, pumping of this level is due to the mechanism of direct transfer of excitation from one molecule to another located in the adjacent node of the chain. In this process, one of the molecules, located on the second excited level, interacting with another molecule in a similar state, gives it its energy, passing into the ground state 1, while the second molecule passes to the third level 3. It is assumed that the third level is inherently electron-vibrational one and decays extremely rapidly with the subsequent energy transfer to the second and first levels. In the resulting system of equations, as a rule, only two-particle interactions are taken into account, which are presented in a factorized form, that is, neglecting correlations between molecules.

However, it is preferable to obtain the system of equations for describing the response of J-aggregates from first principles. In this approach, a hierarchy of interlinking equations arises for the means of the products of operators belonging to different chain molecules. Such a system contains averages, ranging from single-particle and ending with *N*-particle averages (*N* is the number of molecules in the chain), with N>>1.

An important aspect of this problem is that the third level of molecules is a system of a large number of vibrational sublevels, the interaction with which leads to energy dissipation and the irreversibility of the exciton-exciton annihilation process. If, on the basis of first principles, to correctly take into account such interaction, then the equations of motion should be added with a number of multiparticle contributions describing the relaxation of the system associated with exciton-exciton annihilation; these terms are absent, however, within the framework of a purely phenomenological approach. In this case, if we restrict ourselves to considering the equations only for single-particle averages and factorize all the many-particle averages in these equations, then we arrive at the traditional equations [13,19], which now take into account corrections related to three-particle interactions. If we also take into account the system of equations for two-particle averages, then we can thus take into account the pair correlations between the molecules. Further, we will present a derivation of both the refined Bloch equations and the system of equations that takes into account the pair correlations between molecules [23,24,25,26].

Consider a linear chain consisting of *N* three-level molecules. We will assume that the lowest state of each molecule is determined by the state vector |g〉 or |1〉, and the energy of this state is equal $E_{1}$. Accordingly, the second state will be determined by a state vector |e〉 or |2〉 with energy $E_{2}$. The third state is represented by a vector |f〉 or |3〉 with energy $E_{3}$ ($E_{3}>E_{2}>E_{1}$). For energy levels, there is another set of symbols used below, namely: $E_{1}=E_{g},E_{2}=E_{e}$ and $E_{3}=E_{f}$. The molecule located in the node of the chain with the number *m* will correspond to the state vectors |mg〉, |me〉, and |mf〉. Using these vectors for each molecule, we can construct the following creation and annihilation operators for the corresponding states of the molecule: $B_{m}=|mg\rangle \langle me|$—the operator describing the decay of the excitation in the *m* molecule at the “*e*” level and its transition to the ground state, as well as the operator, describing the generation of excitation in the *m* molecule at the level “*e*”. By the same principle, it is possible to define the operators $C_{m}=|mg\rangle \langle mf|$ and $C_{m}^{+}=|mf\rangle \langle mg|$, as well as $D_{m}=|me\rangle \langle mf|$ and $D_{m}^{+}=|mf\rangle \langle me|$. We also introduce the operators of the number of molecules $N_{mg}$, $N_{me}$ and $N_{mf}$ in the states |mg〉, |me〉 and |mf〉, respectively:

(1) $$\{\begin{array}{c} N_{mg}=B_{m}B_{m}^{+}=|mg\rangle \langle me|me\rangle \langle mg|=|mg\rangle \langle mg|,\\ N_{me}=B_{m}^{+}B_{m}=|me\rangle \langle mg|mg\rangle \langle me|=|me\rangle \langle me|,\\ N_{mf}=C_{m}^{+}C_{m}=|mf\rangle \langle mg|mg\rangle \langle mf|=|mf\rangle \langle mf|,\\ N_{mg}+N_{me}+N_{mf}=1. \end{array}$$

All operators belonging to different molecules commute with each other.

The total Hamiltonian of the system consists of the Hamiltonian of free molecules, as well as the Hamiltonians of the interaction of molecules with the external field and with each other. In particular, this Hamiltonian also includes the interaction leading to exciton-exciton annihilation. The interaction Hamiltonian with an external electromagnetic field with a frequency $\nu_{12}$ can be represented in the form

(2) $$H_{el}\left(t\right)=-\frac{1}{2}\sum_{m}\left(\mu^{12}e_{1})(B_{m}^{+}E_{1}\exp(-i\nu_{12}t)+H.c.\right).$$

Here, *H*.*c*. means Hermitian conjugation, the external field is determined by the formula $E=\frac{1}{2}(e_{1}E_{1}\exp(-i\nu_{12}t)+c.c.)$, *c.c*. stands for complex conjugation, the unit vector $e_{1}$ indicates the wave polarization, and the Hamiltonian (2) itself represents the interaction of the specified field with the polarization vector **P** of the molecular system. This interaction is taken into account in the rotating wave approximation, and $\mu^{12}$ is the dipole moment of the molecule for the transition 1→2. In this form, the Hamiltonian of the system is time-dependent. The transition to a stationary Hamiltonian is carried out by an unitary transformation of system operators with the replacement of old operators $B_{m}$ and $D_{m}$ with new ones $b_{m}$ and $d_{m}$:

(3) $$b_{m}=B_{m}\exp\left(i\nu_{12}t\right),\;d_{m}=D_{m}\exp\left(i\nu_{12}t\right).$$

At the same time, the energy of levels in the Hamiltonian of free molecules $H_{0}$ is shifted.

The total Hamiltonian of the system *H* can be represented as the sum of the Hamiltonians composing it

(4) $$H=H_{0}+H_{el}+H_{int}+H_{annih}$$

where the Hamiltonian of free molecules *H_0_*

(5) $$H_{0}=\sum_{m}\left\{\hbar (\omega_{m12}-\nu_{12})\;b_{m}^{+}b_{m}+\sum_{\nu }\hbar (\omega_{m13\nu }-2\nu_{12})\;d_{m\nu }^{+}d_{m\nu }\right\}.$$

In Equation (5) and further, we take into account that the third level of molecules is electron-vibrational and splits into a system of vibrational sublevels, to which the index ν corresponds. Here it is also assumed that $E_{1m}=E_{mg}=0$ and then $\hbar \omega_{m12}$ and $\hbar \omega_{m13\nu }$ are the energies of the second and third levels.

Hamiltonians $H_{el}$, $H_{int}$ and $H_{annih}$ consistently describe the interaction with the pump field, the dipole-dipole interaction (see also [14,27,28,29,30]), and the interaction due to exciton-exciton annihilation. They have the following form:

(6) $$H_{el}=-\frac{1}{2}\sum_{m}\left(\mu^{12}e_{1})(b_{m}^{+}E_{1}+H.c.\right),$$

(7) $$H_{int}=\frac{\hbar }{2}\sum_{k\neq l}\Delta_{lk}\left(b_{l}^{+}b_{k}+b_{l}b_{k}^{+}\right),$$

(8) $$H_{annih}=\sum_{\begin{array}{c} v\\ k\neq l \end{array}}\left(V_{kl}b_{k}d_{l\nu }^{+}+V_{lk}d_{l\nu }b_{k}^{+}\right).$$

Interaction constants are equal [14]

(9) $$\Delta_{lk}=\frac{\mu^{2}}{\hbar a^{3}}\{\left[\frac{\cos(k_{0}a|l-k|)}{|l-k{|}^{3}}+k_{0}a\frac{\sin(k_{0}a|l-k|)}{|l-k{|}^{2}}\right](1-3\cos^{2}\theta )-{\left(k_{0}a\right)}^{2}\frac{\cos(k_{0}a|l-k|)}{\left|l-k\right|}\sin^{2}\theta \}.$$

Here, $\mu =|\mu^{12}|$, $k_{0}$ is the wave vector of the incident radiation, a is the lattice constant, θ is the angle between the direction of the dipole moment μ and the axis of the chain, l,k (l≠k)=1,2,…,N.

#### 2.1.2. Governing Equations

In accordance with the general rules of quantum mechanics, the equation of motion for an arbitrary operator *A* of the system is:

(10) $$\frac{dA}{dt}=\frac{i}{\hbar }\left[H,A\right].$$

It should be noted that the equation of motion thus obtained does not contain interaction through the transverse radiation field that molecules exchange. Since such an interaction contains attenuation associated with radiation, it is usually taken into account directly in the equations of motion by adding a certain type of operator to the right-hand side of Equation (10) [31]. As a result, Equation (10) goes into the equation

(11) $$\frac{dA}{dt}=\frac{i}{\hbar }\left[H,A\right]+2\sum_{l,k}\gamma_{lk}[b_{l}^{+}Ab_{k}-\frac{1}{2}(b_{l}^{+}b_{k}A+Ab_{l}^{+}b_{k})].$$

Here, $\gamma_{lk}$ is given by the formula [14]

(12) $$\gamma_{lk}=\frac{\mu^{2}}{\hbar \;a^{3}}\{\left[k_{0}a\frac{\cos(k_{0}a|l-k|)}{|l-k{|}^{2}}-\frac{\sin(k_{0}a|l-k|)}{|l-k{|}^{3}}\right](1-3\cos^{2}\theta )+{\left(k_{0}a\right)}^{2}\frac{\sin(k_{0}a|l-k|)}{\left|l-k\right|}\sin^{2}\theta \}.$$

As an example, we present the equation obtained on the basis of Equation (11) for the operator of the number of molecules at the second level $N_{me}$ (*m* is the number of the molecule):

(13) $$\frac{dN_{me}}{dt}=i\{-\frac{1}{2\hbar }(\mu^{12}e_{1})E_{1}(b_{m}-b_{m}^{+})+\frac{1}{\hbar }\sum_{n\neq m}\left[\left(\Delta_{mn}+i\;\gamma_{mn})b_{n}^{+}b_{m}-(\Delta_{mn}-i\;\gamma_{mn})b_{m}^{+}b_{n}\right)\right]+\frac{i}{\hbar }\left[H_{annih},N_{me}\right]\}.$$

Physical interest are not in the operators themselves, but in the averages obtained by averaging them over the density matrix of the system. As a result, an infinite system of equations for operators goes into a hierarchical system for the averages of these operators. In this case, the following questions arise: how to make such a system finite and suitable for solution and what minimum set of averages from operators can be limited to an adequate description of the system. If we restrict ourselves to describing the system using averages only from single-particle operators, then in order to close the system of equations, we must present all multi-particle averages entering the equations for single-particle averages as products of the latter. With this description, we neglect the correlations between the molecules. To take into account pair correlations, in addition to single-particle corrections, two-particle averages should also be added. As a result, an additional system of equations arises for these averages. In order to close such a system, multiparticle (the number of particles is more than two) averages must be represented as products of single-particle and two-particle averages. When taking into account the three-particle correlations, the entire process described above is repeated. Thus, the minimum set of operators and their averages, necessary for describing the system, is determined by the accuracy with which we want to describe the interactions between the molecules of the chain. Here we presented the general concept of choosing a base set of averages from system operators.

Taking into account the above, we choose the following operators as a basic set of operators and, therefore, averages from them: $b_{m}$, $N_{me}$, $N_{mf}$, $N_{me}N_{ne}$, $N_{m}b_{n}$, and $b_{m}b_{n}$ (m≠n). This should also include a set of Hermitian-conjugate operators. The physical meaning of the means from these operators will be explained below. By successively substituting the indicated operators as *A* into Equation (11), and then averaging it, we obtain the complete system of equations for describing the chain molecules of the form:

(14) $$\frac{d\langle A\rangle }{dt}=\frac{i}{\hbar }\langle \left[H,A\right]\rangle +2\sum_{l\neq k}\gamma_{lk}[\langle b_{l}^{+}Ab_{k}\rangle -\frac{1}{2}\left(\langle b_{l}^{+}b_{k}A\rangle +\langle Ab_{l}^{+}b_{k}\rangle \right)].$$

The next step is to calculate the relaxation terms associated with the exciton-exciton annihilation and close the resulting system of equations by factoring the many-particle averages, when they are represented as products of averages from the operators in the basic set. The rules of factorization are described in sufficient detail in Reference [23], see also References [27,30,32]. We indicate here only the interpretation of the averages of the operators of the basis set. Thus, the value $\langle b_{m}\rangle$ of this set is proportional to the dipole moment of the molecule *m* for the transition 1→2, $\langle N_{me}\rangle$ and $\langle N_{mf}\rangle$ determine the populations of the second and third levels of the molecule, respectively, and $\langle N_{me}N_{ne}\rangle \;(m\neq n)$ can be associated with the population of two-exciton states. 

The states of the components of the third level of molecules *f* of vibrational sublevels are marked by an index ν and are characterized by a density of states $\delta (E_{f})=\sum_{\nu }\delta (E_{f}-E_{f\nu })$. The system of these sublevels is a reservoir, interacting with which the excited molecule irreversibly relaxes to a lower energy state.

Let us illustrate the calculation of such relaxation for the population of the second level $\langle N_{me}\rangle$. The commutators can be brought to the form:

(15) $$\frac{i}{\hbar }\left[H_{annih},N_{me}\right]=\frac{i}{\hbar }\sum_{\begin{array}{c} v\\ p\neq m \end{array}}[(V_{pm}b_{p}d_{m\nu }^{+}-H.\;c.)+(V_{mp}b_{m}d_{p\nu }^{+}-H.\;c.)].$$

We will assume that the evolution of the operators in (15) associated with the mechanism of exciton-exciton annihilation can be described independently of the effect of all other interactions in the system [33]. Then, for the operator $b_{p}d_{m\nu }^{+}$ we get an equation of the form

(16) $$\frac{d(b_{p}d_{m\nu }^{+})}{dt}=\frac{i}{\hbar }\left[(H_{0}+H_{annih}),b_{p}d_{m\nu }^{+}\right].$$

Averaging Equation (16) over the density matrix of the system and using the Markov process approximation, this equation can be formally solved [23], and the result can be substituted into the averaged Equation (15). As a result, we obtain the expression for the contribution to the relaxation of the system associated with the mechanism of exciton-exciton annihilation:

(17) d〈Nme〉dt=−∑p≠m{wmp〈NmeNpe〉+Re∑k≠pk≠m[(Γpmmk+i2Δpmmk)〈Nmebk+bp〉+(Γmppk+i2Δmppk)〈Npebk+bm〉]}

where constants $\Delta_{pmmk}$, $\Gamma_{pmmk}$, and $w_{mp}$ have the form:

(18) $$\left\{ \begin{array}{l} \Delta_{pmmk}=\frac{1}{\hbar^{2}}P\sum_{\nu }\frac{V_{pm}V_{mk}}{\omega_{fv}-2\omega_{e}},\\ \Gamma_{pmmk}=\frac{2\pi }{\hbar^{2}}\sum_{v}V_{pm}V_{mk}\delta (\omega_{fv}-2\omega_{e}), \end{array}\right.$$

(19) $$w_{mp}=2\Gamma_{mppm}.$$

Averaging Equation (13) and using Equation (17), we obtain the equation for evolution of 〈*N_me_*〉. In order to close the system of equations into which it enters, it is necessary to carry out factorization of many-particle terms in these equations [23]. As a result, we find

(20) d〈Nme〉dt=−γ2〈Nme〉+i{−12ℏ(μ12e1)E1(〈bm〉−〈bm+〉)++1ℏ∑n≠m[(Δm n+i γm n)〈bn+〉〈bm〉−(Δm n−i γm n)〈bm+〉〈bn〉]}−∑l≠m{wml〈NmeNle〉++Re∑k≠lk≠m[(Γlmmk+i2Δlmmk)(〈bk+〉〈Nmebl〉+〈bl〉〈Nmebk+〉−〈bk+〉〈bl〉〈Nme〉)++(Γmllk+i2Δmllk)(〈bk+〉〈Nlebm〉+〈bm〉〈Nlebk+〉−〈bk+〉〈bm〉〈Nle〉)]}+Γ32〈Nmf〉.

In Equation (20), the contribution $\Gamma_{32}\langle N_{mf}\rangle$ associated with the transition from the third to the second level and the rate of transverse relaxation are taken into account phenomenologically. A similar approach is used in the derivation of the equations of motion for other averages included in the basis set. As a result, a closed system of equations arises in which two-particle correlations between molecules are taken into account. Their cumbersome form is given in Reference [23].

In the phenomenological approach of single-particle density matrices for J-aggregate, only two-particle interactions are presented in a factorized form. In the above approach, this corresponds to the system of equations for single-particle averages. However, the analysis of such a system from first principles shows that, in addition to the two-particle contributions, there are also contributions that take into account three-particle interactions. If we factorize the resulting equations, then we arrive at the traditional equations [13,19,24], in which, however, the corrections related to three-particle interactions are taken into account. These corrections arise in the framework of the above approach to the calculation of relaxation terms due to exciton-exciton annihilation. Below is the system of Equations (21)–(24) obtained from Equation (20) and similar equations in this way, in which the additional terms due to three-particle interactions are given in curly brackets. In accordance with Reference [13], the averages of single-particle operators are related to the elements of single-particle density matrices by the relations

(21) $$\langle N_{kf}\rangle =\rho_{33}^{\left(k\right)},\;\langle N_{ke}\rangle =\rho_{22}^{\left(k\right)},\;\langle N_{kg}\rangle =\rho_{11}^{\left(k\right)},\;\langle b_{k}\rangle =\frac{1}{2}R_{k},\;w=2\alpha_{s}.$$

Here, $\rho_{11}^{\left(k\right)}$, $\rho_{22}^{\left(k\right)}$ and $\rho_{33}^{\left(k\right)}$ represent the diagonal elements corresponding to levels 1, 2 and 3 of the molecule with the number *k*, and $R_{k}$ is the non-diagonal element corresponding to the transition 1→2. Then the following Equations (22)–(25) are the result of their factorization taking into account two and three-particle interactions (they occur to be of the same order).

(22) ρ˙22(k)=−12Re[∑l=1,l≠kN(γlk+iΔlk)RlRk*−iΩRk*]+Γ32ρ33(k)−Γ21ρ22(k)−2αsρ22(k)[ρ22(k−1)+ρ22(k+1)]−{14αsRe[Rκ(Rκ−2*ρ22(κ−1)+Rκ+2*ρ22(κ+1))]−14αsρ22(k)(Rk−1*Rk+1+Rk+1*Rk−1)},

(23) $${\dot{\rho }}_{33}^{\left(k\right)}=-\left(\Gamma_{31}+\Gamma_{32}\right)\rho_{33}^{\left(k\right)}+\alpha_{s}\rho_{22}^{\left(k\right)}[\rho_{22}^{\left(k-1\right)}+\rho_{22}^{\left(k+1\right)}]+\left\{\frac{1}{4}\alpha_{s}\rho_{22}^{\left(k\right)}\left(R_{k-1}^{*}R_{k+1}+R_{k+1}^{*}R_{k-1}\right)\right\},$$

(24) $$\begin{array}{l} {\dot{R}}_{k}=-(\Gamma_{⊥}+i\Delta_{k})R_{k}+\sum_{l=1,l\neq k}^{N}\left(\gamma_{lk}+i\Delta_{lk})R_{l}[\rho_{22}^{\left(k\right)}-\rho_{11}^{\left(k\right)}\right]-i\Omega [\rho_{22}^{\left(k\right)}-\rho_{11}^{\left(k\right)}]-\alpha_{s}R_{k}[\rho_{22}^{\left(k-1\right)}+\rho_{22}^{\left(k+1\right)}]-\\ \left\{\frac{1}{2}\alpha_{s}\left(R_{k-2}\rho_{22}^{\left(k-1\right)}+R_{k+2}\rho_{22}^{\left(k+1\right)}\right)\left(1-2\rho_{22}^{\left(k\right)}-\rho_{33}^{\left(k\right)}\right)-\frac{1}{8}\alpha_{s}R_{k}\left(R_{k-1}^{*}R_{k+1}+R_{k+1}^{*}R_{k-1}\right)\right\}, \end{array}$$

(25) $$\rho_{11}^{\left(k\right)}=1-\rho_{22}^{\left(k\right)}-\rho_{33}^{\left(k\right)}.$$

Here, $\alpha_{s}$ is the rate of exciton-exciton annihilation, $\Omega =\mu^{12}E/\hbar$ is the Rabi frequency, *E* is the field amplitude, $\Gamma_{m\;n}$ is the rate of relaxation from level *m* to level *n*, $\Gamma_{⊥}$ is the transverse relaxation rate of the molecule, $\Delta_{k}\equiv \Delta$ is the detuning of the frequency of radiation from the resonance for an isolated molecule.

#### 2.1.3. Bistability for Molecular J-Aggregates

Bistability means that, for the same parameters, it has two possible states with different characteristics. Here we will consider homogeneous, along the chain, distributions of excitation under homogeneous irradiation of the chain. The dynamics of an infinite homogeneous molecular chain is described by the system of Equations (22)–(25), assuming that all elements of the density matrix do not depend on the number of the molecule. The stationary states of the chains in this system correspond to zero time derivatives. The bistability follows already from the simplest version of these equations without taking into account multiparticle terms [13,19]. In this Section, we analyze the effect of many-particle phenomena on the manifestation of bistability. The analysis here is performed irrespective of the stability of solutions in the presence of small perturbations; the latter is studied in the next Section.

**Figure 1:**
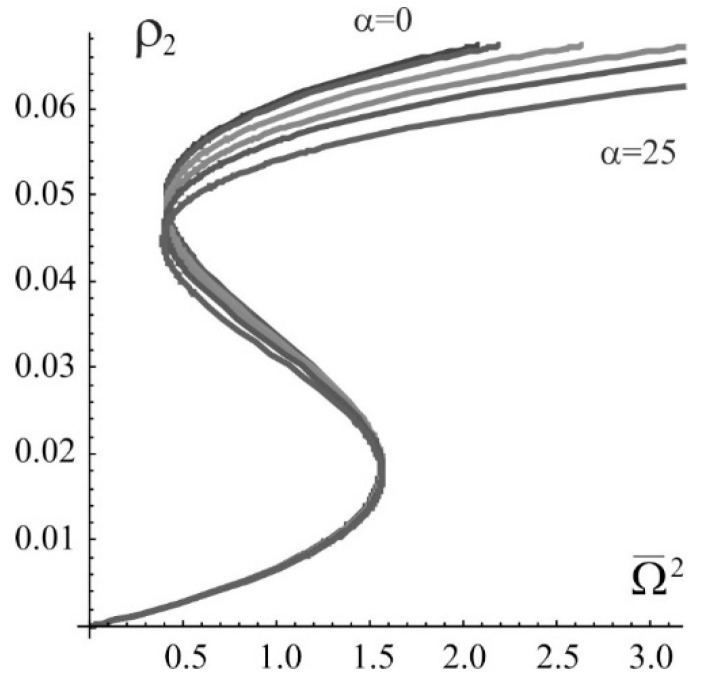
The bistability of the stationary population of the second excited level with a change in the Rabi frequency without taking into account the many-particle corrections. The coefficient of exciton-exciton annihilation takes the values: α=0, 1, 5, 10, 15, and 25; frequency detuning $\bar{\;\Delta }=-10.$

**Figure 2:**
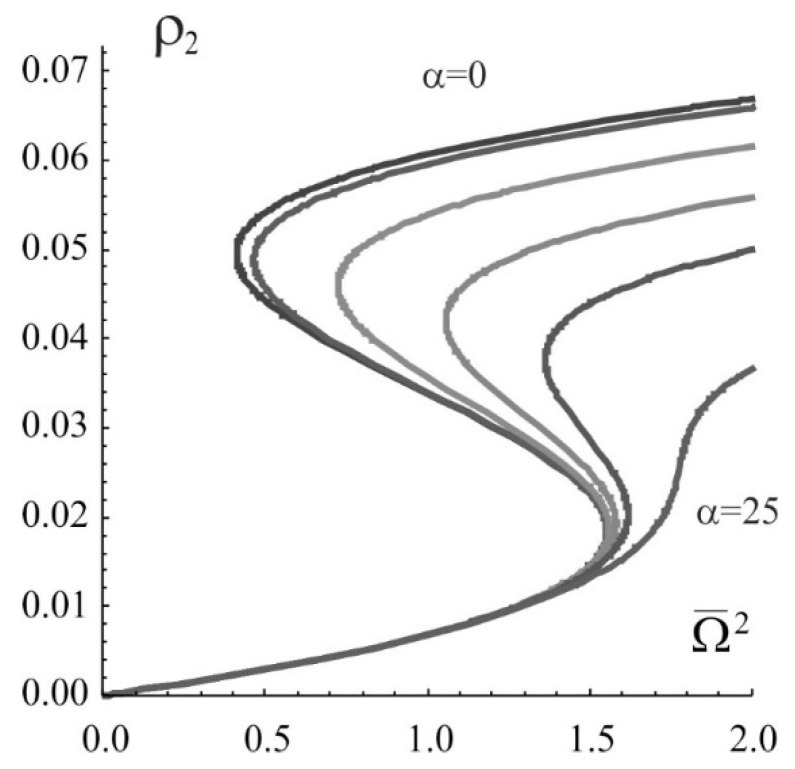
Bistable dependence of the population of the second (excited) level on the Rabi frequency for stationary homogeneous states.

**Figure 3:**
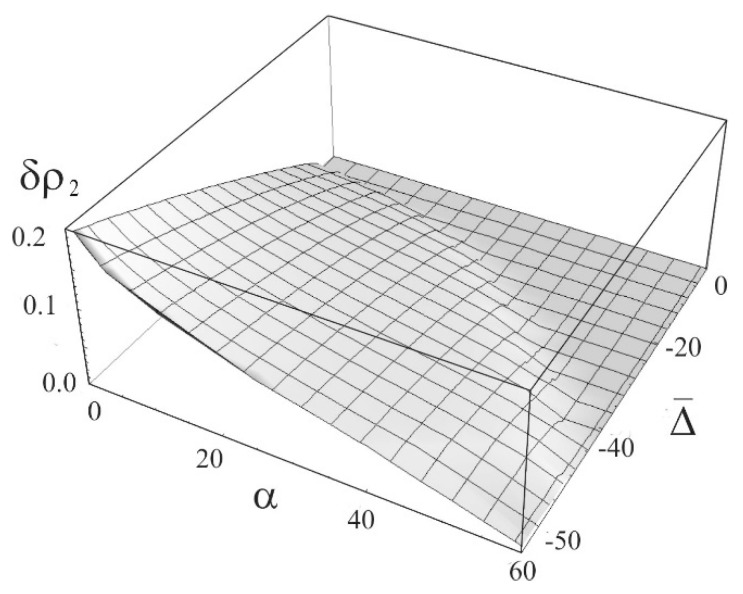
Dependence of the width of the bistability region of the population of the second level on the coefficient of exciton-exciton annihilation α and detuning $\bar{\Delta }$.

Excluding the many-particle effects, the effect of the rate of exciton-exciton annihilation on the width of the bistability region is shown in Figure 1 in the form of the dependence of the stationary population of the second (excited) level $\rho_{2}=\rho_{22}$ on the normalized radiation intensity ${\bar{\Omega }}^{2}$ at normalized detuning $\bar{\Delta }=-10$ and α = 0, 1, 5, 10, 15, and 25. Similar dependence when taking into account three-particle effects is given in Figure 2. One can see dramatic difference, especially at high rates of annihilation. With an increase in the rate of exciton – exciton annihilation α, the bistability region narrows and disappears at α≃22.7. In Figure 3 shown is the dependence of the width of the bistability region $\delta \rho_{2}$ (the difference in the values of the population of the second level on the right and left boundaries of bistability in Figure 2) on the detuning and rate of the exciton-exciton annihilation. The result makes it possible to determine not only the region of existence of the bistability itself, but also the most probable region of parameters in which a dissipative soliton can be formed.

#### 2.1.4. Stability of Homogeneous Distributions

This Section presents a linear stability analysis of the stationary homogeneous states of the chains studied in the previous section. The initial one is the system of Equations (22)–(25). Solutions are written in the form of a stationary homogeneous solution (superscript 0) and perturbations with a small amplitude $x_{n}$:

(26) {ρ33(k)=ρ330+12(x1eλt+ikq+c.c.),ρ22(k)=ρ220+12(x2eλt+ikq+c.c.),ReRk=R0R+12(x3eλt+ikq+c.c.),ImRk=R0I+12(x4eλt+ikq+c.c.).

Here, *c.c*. means complex conjugation. The parameter *q* can be considered as the normalized wave number of the perturbation. Instability arises if the maximum value of the increase in the perturbation becomes positive when *q* varies, maxReλ>0. For a fixed *q*, the value λ is determined from a fourth degree algebraic equation with real coefficients.

**Figure 4:**
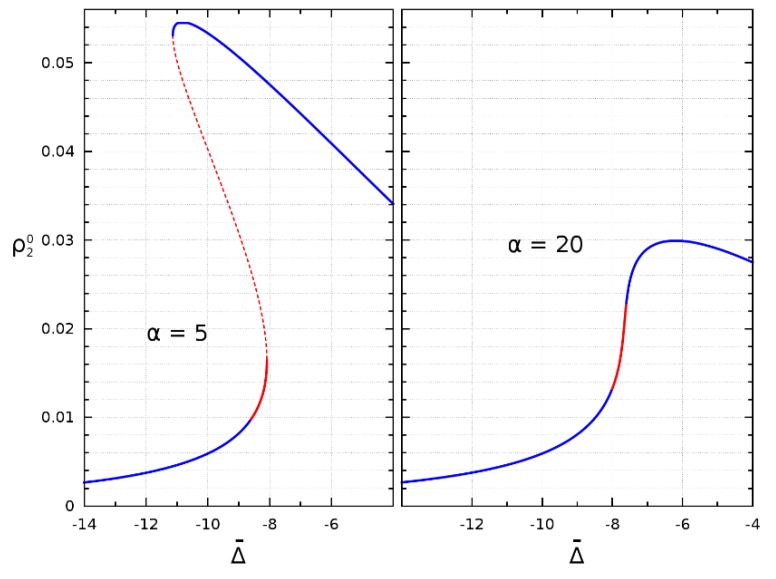
The population of the second excited level as a function of frequency detuning for α=5 (bistability) and 20 (monostability) and $\bar{\Omega }=1$. The solid blue lines correspond to the stability, the dashed red line corresponds to the unstable intermediate branch, and the solid red line corresponds to the instability region on the lower branch (left) or in the case of monostability (right).

The states corresponding to the intermediate branch of a bistable dependence are obviously unstable. The analysis shows that the states corresponding to the upper branch of this dependence are stable throughout the entire parameter range studied. Finally, the modes corresponding to some parts of the lower branch of the bistable dependence, as well as some of the states in the monostability mode (Figure 4), turn out to be unstable.

#### 2.1.5. Discrete Switching Waves and Dissipative Molecular Solitons

In this Section, we move from uniform distributions of the excitation of the J-aggregate, possible only in an idealized model of an unrestricted chain, to spatially non-uniform, which are of the greatest scientific and applied interest. Namely, on the basis of [18,19,20,21,22], two main regimes are considered: Molecular switching waves and dissipative solitons characterized by nanometer sizes.

We consider J-aggregates of cyanine dyes (PIC:Cl)*_N_* consisting of *N* molecules of pseudo-isocyanine chloride (PIC:Cl) where the value *N* can reach hundreds and thousands. For moderate *N*, the aggregate stable geometry, energies and intensities of lowest singlet electronic transitions can be found by quantum-chemistry methods [32]. Here, as well in the previous Sections, we model J-aggregate with a linear or circular chain of *N* three-level molecules each of them interacting with laser radiation and with other molecules via radiation emitted by molecules. Two lowest electronic levels 1 and 2 form an optical transition in quasi-resonance with the laser radiation frequency $\omega_{0}$. The third level is introduced to describe annihilation of excitations on two neighboring molecules: One of them is deactivated, and the other is activated third level with subsequent relaxation to the second (2) or ground (1) state. The frequency of transition from the ground to the third state is close to double frequency of the main transition 1→2, i.e., $\omega_{31}\approx 2\omega_{21}$. The intermolecular distance *a* is much less than the radiation wavelength, $\lambda_{0}=2\pi c/\omega_{0}$, where *c* is the light speed in vacuum. The laser radiation is linearly polarized. The governing equations were introduced in the previous sections; here we use in simulations their simplified version neglecting terms corresponding to many-particle interactions.

• Ring aggregates

An advantage of such geometry of the molecular chain is the possibility to avoid effect of its ends. Similar to the case of macroscopic spatially distributed optical bistable systems [34,35], under conditions of stability of two homogeneous states, switching waves can be formed—fronts between the two asymptotically homogeneous states. The front can move with constant velocity depending on the intensity of laser radiation. More precisely, switching waves in discrete systems have certain specificity, e.g., the front velocity vanishes not at a unique intensity value (as in continuous systems), but in a certain range of intensity. However, for the parameters used here for J-aggregates, the discreteness does not manifest itself essentially.

**Figure 5:**
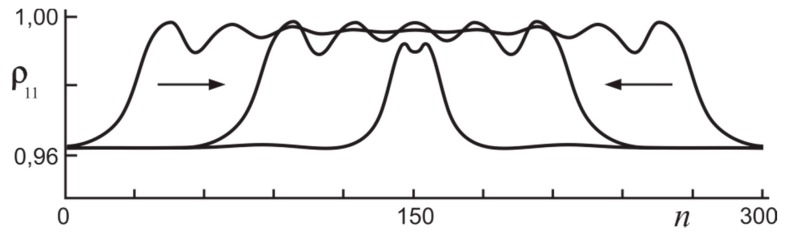
The dynamics of the ground state population in the regime of collision of a pair of counter-propagating switching waves. Arrows show the direction of fronts of switching waves propagation. Time moments *t* = 0 (the widest distribution), 120 and 440 (stable soliton, the narrowest distribution); N=300, $\bar{\Omega }=0.95$.

To form switching waves under conditions of “classical” bistability–bistability of homogeneous states, it is sufficient to create the initial condition corresponding to one of the homogeneous states over one part of the aggregate chain, and the other homogeneous state over the rest part of the chain. In further evolution, two switching waves form whose example is given in Figure 5. Here one can see propagation of two counter-propagating switching waves that do not interact one with other while the distance between the fronts essentially exceeds the front width. However, fronts of counter propagating switching waves approach one to the other with time, and finally they form a stable soliton illustrated by the narrowest distribution in Figure 5.

**Figure 6:**
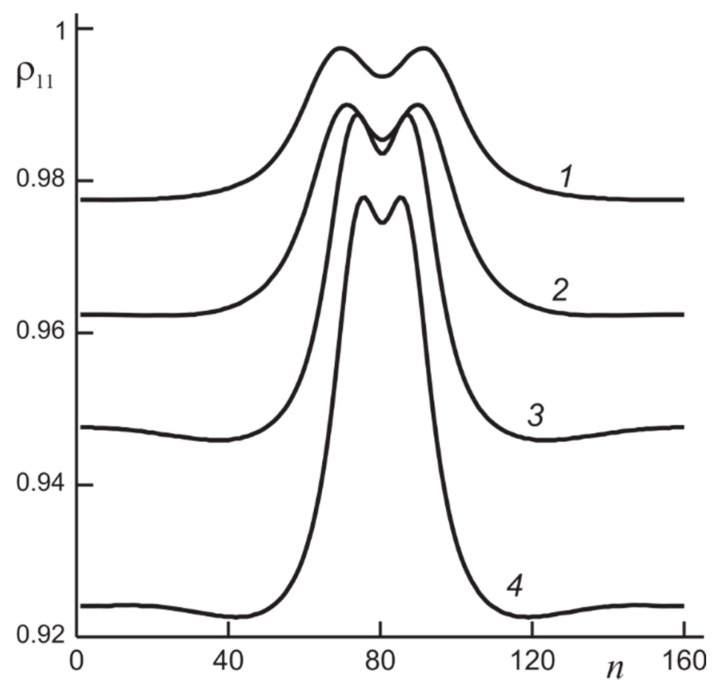
Profiles of the ground state population for stationary solitons for the Rabi frequency $\bar{\Omega }=0.45$ (1), 0.48 (2), 0.78 (3), and 0.95 (4).

Important is that the initially wide population inhomogeneity shrinks gradually to a narrow one and finally can stabilize. For the parameters used in the simulation [19,20,21], the collision of counter-propagating switching waves results in formation of a stable localized structure—a molecular dissipative soliton. This is one of the main scenarios of such dissipative solitons formation; in another one, the stability of two branches of bistability is not required. The region of parameters in which solitons exist is narrower than the region of bistability. With parameters change, features of solitons, as well as those of the switching waves, change. In Figure 6, profiles of population of the ground state for solitons for a number of values of the Rabi frequency.

• Linear aggregates

**Figure 7:**
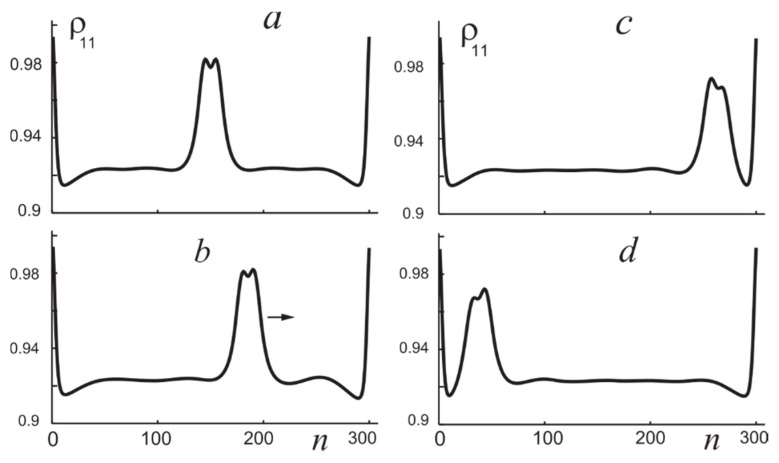
Profiles of population of the excited state for time moments t=0 (**a**), 50 (**b**), 300 (**c**), and 700 (**d**), see the text.

**Figure 8:**
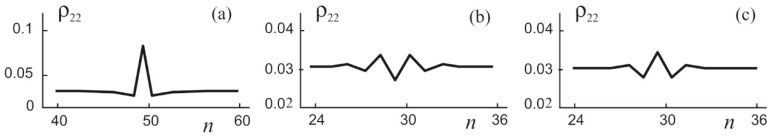
Profiles of the excited level population in the regime of narrow solitons.

For linear chains with finite number of molecules, edge effects become essential, and we can speak of solitons only if the length of the chain significantly exceeds the soliton size. Even if this condition is fulfilled, the steady-state position of the soliton in the chain is not arbitrary: with time, it moves to the center of the chain. The position and motion of a soliton in a chain can be controlled by changing the shape of the radiation incident on the chain. For example, an oblique incidence of radiation induces the soliton motion, the direction of which is determined by the gradient of the radiation phase (inclination of the wave). We illustrate this by Figure 7 for the linear chain of 300 molecules. The initial position of soliton for normal incidence is in the chain centre (a, t=0). Then we change the radiation angle of incidence to the value φ=0.1 (b, t=50). At this stage, the soliton propagates along the chain towards to the right edge (b) and stops near it (c, t=300). After that, angle of incidence changes its sign, φ=−0.1. Then the soliton propagates towards the chain left edge and stops there (d, *t* = 700). Modulating profiles of the phase and intensity of the incident radiation, it is possible to form several solitons and to control their position if the number of molecules in the chain is sufficiently large. More exactly, it is necessary to ensure a sufficiently small statistical dispersion of the frequencies of the main electron transition in the molecules, which is caused by their interaction with a random environment [19]. Variation of parameters allows one to find extremely narrow molecular solitons (Figure 8), and it is important to take into account effects of multiparticle interactions and correlations for reliable definition of corresponding parameter ranges.

### 2.2. Organic Thin Films: Bistability and Switching Waves

In References [3,4], purely organic materials were demonstrated with low losses and controllable “plasmonic” features promising for various applications: large enhancement of non-linear optical processes, super-resolution imaging, optical cloaking, etc. [1,36,37,38]. The theory of these features was developed in Reference [39] for two-level scheme of organic molecules interacting with laser radiation. The frequency of transition 1→2 between electronic levels 1 (ground state) and 2 (excited state) is close to the frequency of laser radiation ω. According to References [39,40], the excited state population $n_{2}$ dynamics in homogeneous mode, regardless to its stability, is described by the balance equation

(27) $$\frac{dn_{2}}{dt}=-F(n_{2}).$$

Here

(28) $$F(n_{2})=\frac{n_{2}}{T_{2}}\left\{1+\left[1+\exp\left(-\frac{\hbar (\omega_{st}-2\delta \omega )}{k_{B}T}\right)\right]\exp\left(-\frac{\delta \omega^{2}}{2\sigma_{2s}}\right)J\right\}-\exp\left(-\frac{\delta \omega^{2}}{2\sigma_{2s}}\right)J,$$

$T_{2}$ is the lifetime of the excited state, $\omega_{st}=\hbar \sigma_{2s}/(k_{B}T)$, $\sigma_{2s}$ is the second central moment of an absorption spectrum, $k_{B}$ is the Boltzmann constant, T is the temperature, $\delta \omega =(\omega_{21}-\omega )-p(1-2n_{2})$, $\omega_{21}$ is the frequency of Franck-Condon transition 1→2, $p=[4\pi /\left(3\hbar \right)]|\mu^{12}{|}^{2}N$ is the strength of the near dipole-dipole interaction, $\mu^{12}$ is the dipole moment of the molecule for the electronic transition 1→2, as well as for J-aggregates, N is the density of molecules, $J=\sigma_{a}T_{2}[{\left(\varepsilon_{b}+2\right)}^{2}/9]J_{l}$ is normalized laser intensity, $\varepsilon_{b}$ is the “bulk” relative permittivity, and $J_{l}$ is laser intensity. Important is that the function $F(n_{2})$ depends linearly on the radiation intensity *J*.

The analysis shows that it is possible to realize a bistable dependence of populations of levels on laser intensity in the case of dense medium at the blue side of the absorption spectrum [41]. Therefore it is natural to expect existence of switching waves in organic films of sufficient size. In contrast with the previous Sections, the model is now continuous one (not discrete). In the framework of phenomenological diffusional approach [42] the dynamics of the film with a strip form is described by the following generalization of Equation (27):

(29) $$\frac{\partial n_{2}}{\partial t}=D\frac{\partial^{2}n_{2}}{\partial x^{2}}-F(n_{2}).$$

Here, $D=0.171(p^{2}/N^{2/3})T^{\prime }$ is the diffusion coefficient and $T^{\prime }$ is the irreversible dephasing time of the electronic transition [43]. Under homogeneous irradiation, there are stable stationary homogeneous solutions of Equation (29). Therefore bistable mode of thin organic films is indeed available. 

**Figure 9:**
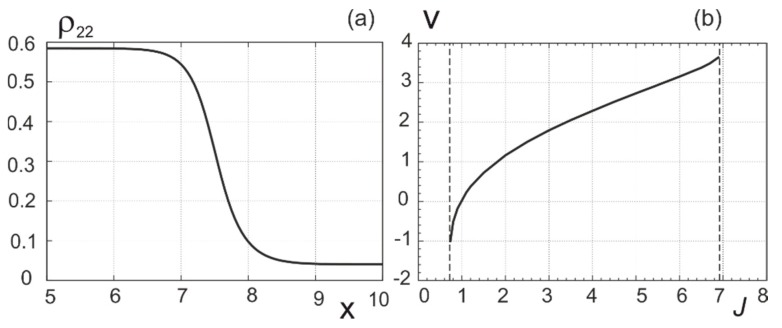
Instantaneous profile of population of excited state in the mode of switching wave (**a**) and dependence of the wave velocity on the radiation intensity (**b**).

In the framework of Equation (29), solitons do not exist under film uniform illumination. Nevertheless switching waves can be formed in organic films with sufficient sizes. In Figure 9, we present the typical shape of their front and the velocity of its motion. The velocity changes sign with variation of radiation intensity, and the switching wave front stops at some (“Maxwellian”) value of radiation intensity. The maximum velocity of switching waves $v_{\max}∼\sqrt{D/T_{2}}∼10^{5}$ cm/s ($T_{2}∼10^{-9}$ s). The film size should exceed the width of the switching wave front $∼\sqrt{DT_{2}}∼1$ μm. Note also that it is possible to support solitons if radiation intensity is spatially modulated [35].

## 3. Light Structuring

The structuring of light has become possible at present, mainly due to the progress in laser physics and technology. The previous Section presents the prospects for generation in nano-structured media of extremely concentrated light structures. The ability to control the shape of the wavefront of light with the use of space-time modulators and holographic equipment [44] allows us to raise the question of the formation of stable packages of light with a nontrivial internal structure localized in all three spatial dimensions and in time. The interest in 3D-topological dissipative optical solitons is caused both by the richness and uncommonness of their properties, and by their increased stability and potential for informational applications. Indeed, in a homogeneous nonlinear dissipative medium of sufficiently large dimensions, a large number of such solitons can be formed, and when coding information with topological indices, essential is their preservation even with significant distortions in the system or variation in its parameters. The limits of permissible variations determine the working range of this approach. 

Topological conservative knot solitons were pioneered by Faddeev in classical field theory [45,46], and currently they are known in a wide circle of physical systems including Bose-Einstein condensates and superconductors [47,48]. Note also that standard conservative saturable nonlinearities do not suppress azimuthal instabilities of vortex solitons [49], but competing nonlinearities have been predicted to support such stable solitons in both 2D [50,51] and 3D [52,53] geometries. 

Related complex cubic-quintic Ginzburg–Landau models give rise to stable 3D fundamental solitons [54] and vortex tori [55]; however these models connection with laser schemes is not evident. Recently, dissipative mode-locked cavity solitons with a pulse duration shorter than the cavity round trip, which may be considered as isolated 3D objects, were observed in a vertical- cavity surface- emitting laser [56]. Such dissipative settings are promising for the generation of multidimensional solitons because they appear as stable attractors with a broad attraction basin, a property that should facilitate their creation. These approaches are analyzed in a recent review [57].

This Section is devoted to the analysis of this issue basing on the works [58,59,60,61,62] using the previous research of dissipative solitons reviewed in [22].

### 3.1. Model of a Laser with Saturable Absorption and Governing Equations

**Figure 10:**
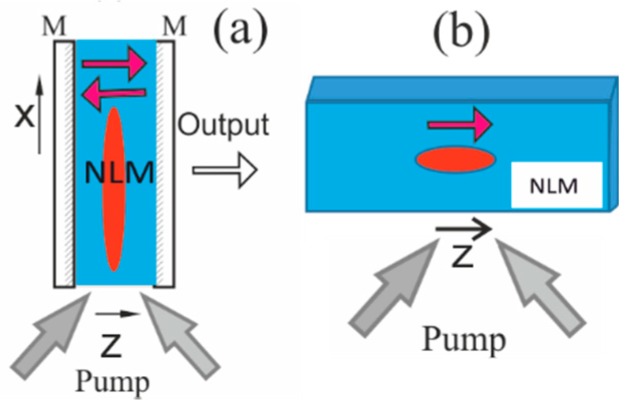
The schemes supporting topological laser solitons. (**a**) A wide-aperture laser with saturable absorber and mirrors M; *x* is a transverse Cartesian coordinate. (**b**) Cavityless scheme. NLM is nonlinear medium with saturable amplification and absorption, pump is incoherent.

We will consider a homogeneous medium with fast saturable laser amplification and absorption (Figure 10). Such a model is appropriate also for a wide-aperture laser with sufficiently long ring cavity (the round trip time exceeds relaxation rates of the medium). The medium consists of a linear matrix with non-resonance absorption and frequency dispersion, and inserted into the matrix centers of two types: active (with laser gain) and passive (with saturable absorption). The centers can be realized as nanoobjects–quantum dots with two levels, the transition between which is resonant to laser radiation. The relaxation rates of the levels are shorter than the laser pulse duration. As well as in Section 2, laser radiation packet (beam and pulse) propagating though the medium is close to a plane monochromatic wave with linear polarization. 

Under approximation of slowly varying electric field envelope *E*, the governing equation is the generalized complex Ginzburg-Landau equation (in dimensionless form) [58,59,60,61,62]:

(30) $$\frac{\partial E}{\partial z}=\sum_{n=1}^{3}c_{n}\frac{\partial^{2}E}{\partial x_{n}^{2}}+f(|E{|}^{2})E.$$

Here *z* is the longitudinal coordinate along the main direction of radiation propagation (evolution variable), $x_{1,2}$ are the transverse Cartesian coordinates, $x_{3}=\tau =t-z/v_{g}$ is time in the accompanying coordinate system moving along *z* with the group velocity $v_{g}$. Terms with coefficients $c_{n}=i+d_{n}$, $d_{n}\geq 0$ describe diffraction ($Re\;c_{n}$, n=1,2), dispersion ($Re\;c_{3}$), and finite width of line of amplification and absorption (Im c3). The linear terms with Im c1,2 corresponds to the dependence of losses on the direction of wave propagation; their connection with non-resonant absorption is indicated below. In general case, function *f* of intensity $I=|E{|}^{2}$ is complex, but for small frequency detuning it is real:

(31) $$f(I)=\frac{g_{0}}{1+I/\beta }-\frac{a_{0}}{1+I}-1.$$

The first term in the RHS of Equation (31) describes the saturable amplification, the second is saturable absorption, and the last is the coefficient of non-resonance losses (after normalization of *z*); $g_{0}$ is the coefficient of small-signal gain, $a_{0}$ is the same for resonance absorption, and *β* is the ration of saturation intensities for gain and absorption. 

Now let us comment on the physical sense of the terms with Im c1,2 [63]. Due to their linear origin, it is sufficient to start with the linear scalar Helmholz equation for the monochromatic electric field with frequency ω (the harmonic time dependence is omitted, the full electric field is given by Re[E˜exp(−iωt)]e where e is the unit vector indicating radiation polarization):

(32) $$\Delta \tilde{E}+k^{2}\tilde{E}=0.$$

Here, $\Delta =\frac{\partial^{2}}{\partial x^{2}}+\frac{\partial^{2}}{\partial y^{2}}+\frac{\partial^{2}}{\partial z^{2}}$ is the Laplace operator and $k=\frac{\omega }{c}n$ – complex wave number; $n=n^{\prime }+in^{\prime \prime }$ is complex refractive index, c is the light velocity in vacuum, k′=Rek=ωcn′, k″=Imk=ωcn″. We consider the case n′=Ren>0, n″=Imn>0 and $n^{\prime \prime }<<n^{\prime }$ (media with weak absorption). The radiation beam propagates predominantly normally to the layer of the media (along z), the layer interfaces are with antireflection coating. In the standard approach, the envelope E is introduced by the ansatz E˜=Re[Eexp(ik′z)]. Then, taking into account slow variation of E as compared with the exponent, we reduce Equation (32) to the following equation:

(33) $$2ik^{\prime }\frac{\partial E}{\partial z}+\Delta_{⊥}E+2ik^{\prime }k^{\prime \prime }E=0,$$

where $\Delta_{⊥}=\frac{\partial^{2}}{\partial x^{2}}+\frac{\partial^{2}}{\partial y^{2}}$ is the transverse Laplace operator. The next ansatz $E=E_{0}\exp(-k^{\prime \prime }z)$ removes the last term in the LHS of Equation (33) and we come to the standard quasioptical equation

(34) $$2ik^{\prime }\frac{\partial E_{0}}{\partial z}+\Delta_{⊥}E_{0}=0.$$

In such a wave, we ignore the difference in absorption of waves propagating, depending on the direction of propagation, along a longer distance in the layer of absorbing medium. However, we can take into account this factor with different ansatz: E˜=Re[Eexp(ikz)]. Then, we get from Equation (32) the equation coinciding with Equation (34) with the replacement $k^{\prime }\to k=k^{\prime }(1+id)$; here, $d=\frac{n^{\prime \prime }}{n^{\prime }}$ (0<d<<1) is the “effective diffusion coefficient” describing angular selectivity of losses and corresponding to coefficient Im c1,2 in Equation (30). Dividing this equation on the term (1+id), taking into account the smallness of d, one obtains the following final equation:

(35) $$2ik^{\prime }\frac{\partial E}{\partial z}+\left(1-id\right)\Delta_{⊥}E=0.$$

It corresponds to increase of optical path and therefore losses with increase of angle between the direction of propagation of the wave (or a ray) and the normal to the layer of absorbing medium.

### 3.2. Topological Laser Solitons

The 3D-topological solitons are obtained by solution of governing Equation (30) with the initial condition using some manipulations–rotation and twist–with investigated earlier [22] 2D-vortex solitons as indicated in [59]. As other dissipative optical solitons, they reflect the dynamical balance of energy input and output. Important role for them plays the “transverse” electromagnetic energy flow (the Poynting vector, additional to the main “longitudinal” one in the direction z)

(36) S=Im(E*∇3E)=I∇3Φ,

where Φ is radiation phase [$E=I^{1/2}\exp(i\Phi )$] and gradient $\nabla_{3}$ acts in the 3D-space $r_{3}=(x,y,\tau )$. In accordance with Equation (36), the energy flow is directed along the phase gradient and is proportional to radiation intensity *I*. The sign of $\mathrm{div}\;S(r_{3})$ determines whether point $r_{3}=(x,y,\tau )$ is a source of electromagnetic energy ($\mathrm{div}\;S(r_{3})>0$) or a sink ($\mathrm{div}\;S(r_{3})<0$).

The key element of topological optical structures are vortex lines where the field vanishes (E=0) and radiation phase changes by 2πm when walking around the line along a closed contour near it in a plane orthogonal to the line tangent. The integer *m* is the topological index. Under our conditions, only the vortex lines with a single topological charge are stable (the case of m=0 is excluded, since it does not correspond to phase singularities). We choose the direction of orientation of the line so that it is m=1.

Geometrically, a vortex line can be closed or unclosed; it geometry is characterized by the accompanying trihedron. In dissipative systems, the energetic characteristics are equally important. Indeed, since the intensity of the radiation on the line itself vanishes, the Poynting vector on it S=0. However, near the line S≠0 and has a predominantly azimuthal component (energy vortex around the vortex line). At the same time, there is also a smaller tangential component of the energy flow near and along the line. A vortex line will be called non-alternating if the sign of the tangential component is preserved along its entire length, and alternating if this sign changes along the line [60]. The points on the line of change of this sign are special. Vortex lines are attractors for neighboring lines of energy flow, such that the tangent at any point **r** on these lines is parallel to **S**: $[\nabla_{3}r\times S]=0$.

Near the line, with the possible exception of its singular points, the radiation intensity is close to axisymmetric, so that in local cylindrical coordinates (ρ,φ,l)

(37) $$E(x,y,l)=a(\rho ,l)\exp(i\varphi +i\Phi_{r}).$$

Module *l* is compared with the length of the energy flow line directed along **S**. The length of the curve is measured from its arbitrary point. The amplitudes *a* and the full phase Φ are considered to be real, the latter being represented as the sum of the azimuthal and radial (depending on *l*) phases:

(38) $$\Phi (\rho ,\varphi ,l)=\varphi +\Phi_{r}(\rho ,l).$$

Unclosed vortex lines at their periphery approaches asymptotically to straight lines. They are alternating because far from laser localized structures the energy flow is directed towards periphery. As will be shown later, closed vortex lines can be both alternating and nonalternating.

On the vortex line itself, the full phase is not defined, contrary to the radial phase $\Phi_{r}$. If we compare the gradient of the full phase Φ to velocity $v=\nabla_{3}\Phi$, then the vector field of velocity is irrotational ($\nabla_{3}\times v=0$) everywhere, except for the vortex lines, where there is a singularity of the delta function type. In accordance with the definition of a topological charge, the phase change when walking along a circuit around the vortex line δΦ=∮v⋅dl=2πm.

**Figure 11:**
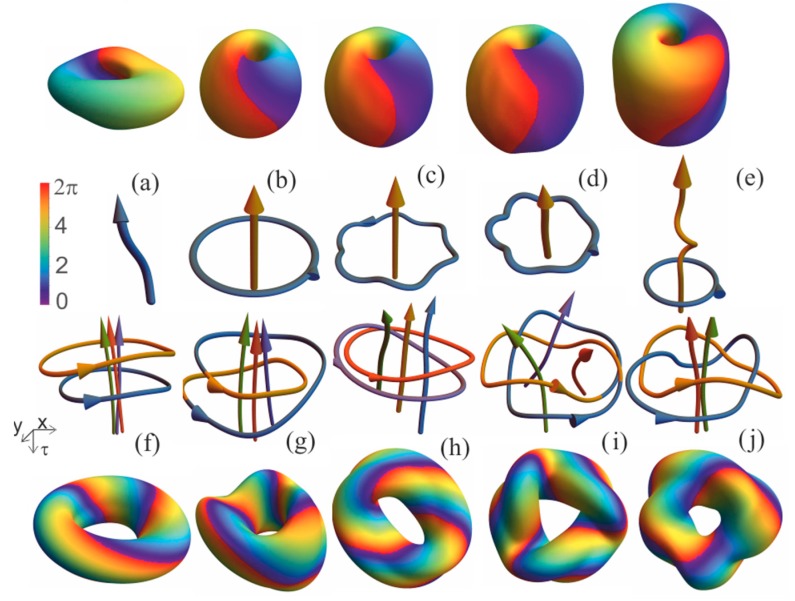
The upper and lower rows: isointensity surfaces of tangle laser solitons at intensity level $0.5I_{\max}$. Two middle rows: Skeletons of the solitons–arrays of their vortex lines. The number of unclosed vortex lines is one (**a**–**e**), two (**j**) and three (**f**–**i**). Unclosed lines (**a**) are absent, “precesson”, (**b**)–(**e**)—one unknotted closed, “apples”, (**f**)—two knotless unlinked, (**g**)—one, trivial knot, (**h**)—two unknotted, a single Hopf link, (**i**)—one knotted, a trefoil knot, (**j**)—two unknotted, with a double link, “Solomon link”. The torsion index of the closed lines s=0 (**b**–**f**), —1 (**g**), —2 (**h**), —3 (**i**), —4 (**j**). The arrows on the vortex lines indicate the direction of the increasing phase of the radiation (m=1 ).

A collection of topological laser solitons found in [58,59,60,61,62,64] is presented in Figure 11. The upper and lower rows are isointensity surfaces, which characterize the external dimensions of topological solitons. Their internal structure is more clearly revealed by the two middle rows where depicted are the "skeletons" of solitons—the sets of their vortex lines. These skeletons are topological tangles [65] because they consist of closed and unclosed lines.

Presented tangle solitons are characterized by a number of topological characteristics. First, there are numbers of unclosed $N_{U}$ and closed $N_{C}$ vortex lines (all vortex lines mentioned below have topological charge m=1). Next, we use a parent 2D-soliton [22] with $N_{v}$ vortices (with unit topological charges), which can have an axis of symmetry of order $N_{s}$ (the distributions of intensity and energy flux do not change when rotating in this plane around the center of symmetry by an angle of $2\pi /N_{s}$). Then we rotate this 2D distribution around an axis lying in the same plane on a full turn with simultaneous twist on angle $2\pi s/N_{s}$. Traces of 2D-vortices are fragments of 3D-spirals. After one turn, an initial 2D-vortex may arrive at the same or some other of the $N_{v}$ 2D-vortices. Correspondingly, the fragments of 3D-spriral form, after possible joining, $N_{C}$ closed vortex lines ($N_{C}\leq N_{v}$). Finally, we introduce several straight vortex lines codirected to the axis and placed inside the spirals, so that the resulting number of unclosed (straight) vortex lines is equal to $N_{U}$. These values—the order of 2D-symmetry $N_{s}$, numbers of unclosed *N_U_* and closed $N_{C}$ vortex lines, and the torsion index of the closed lines s—fully characterize the topological structure of solitons presented in Figure 11. Alternatively, the structure may be characterized by phase incursions while circling the vortex lines and along them. These indices can also be connected with the traditional form of topological indices, including linking number [65].

The scheme described above can be consider as the initial data for study of next dynamics according to Equation (30) (here we use in simulation the simplest case $c_{n}=i+d$, 0<d<<1). If after sufficiently long transient we get a soliton whose structure is topologically equivalent to the initial one, we can characterize the soliton by indices of the initial structure.

Now let us give some comments on Figure 11. The “precesson” (Figure 11a) has only one unclosed vortex line, $N_{U}=1$, $N_{C}=0$, and its skeleton is topologically equivalent to one straight line with charge m=1. The parent 2D-structure for precesson is a fundamental 2D-laser soliton with axial symmetry ($N_{s}=\infty$). All “apple” solitons (Figure 11b–e) with $N_{U}=1$, $N_{C}=1$, are equivalent to that shown in Figure 11b: one straight line with one circle around the line. The 2D-parent structure is 2D-vortex soliton (m=1,$N_{s}=\infty$). Despite the equivalence of topology, these types of “apple” solitons differ both quantitatively and qualitatively, as will be illustrated below. All other solitons shown in Figure 11f–j, have such a 2D-parent as a pair of two strongly coupled vortex solitons with m=1 and $N_{s}=2$. All other indices are given in capture to Figure 11.

**Figure 12:**
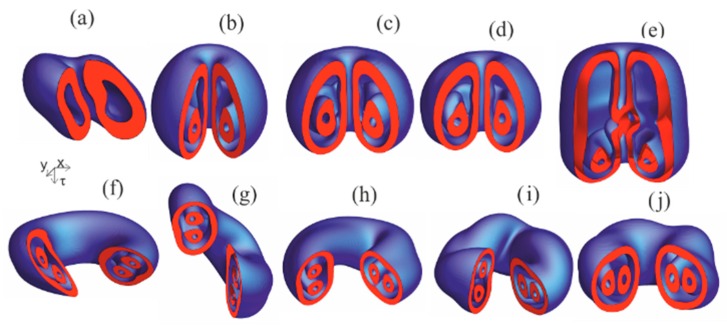
Domains of electromagnetic energy sources (red) and sinks (blue) for solitons shown in Figure 11 with the same labels.

Some insight on the soliton internal structure gives the scalar field of the Poynting vector divergence div **S**. A point **r**_3_ corresponds to energy source if div **S**(**r**_3_) > 0 and to energy sink if div **S**(**r**_3_) < 0. Surfaces div **S**(**r**_3_) = 0 separate domains of energy sources and energy sinks of toroidal shape, see Figure 12.

More detail for laser soliton internal structure gives analysis of the vector field of electromagnetic energy flow (the Poynting vector **S**). Lines of energy flow are determined by the system of three ordinary differential equations of the first order:

(39) $$dx_{j}/dl=S_{j}(r_{3}),\;z=const.$$

**Figure 13:**
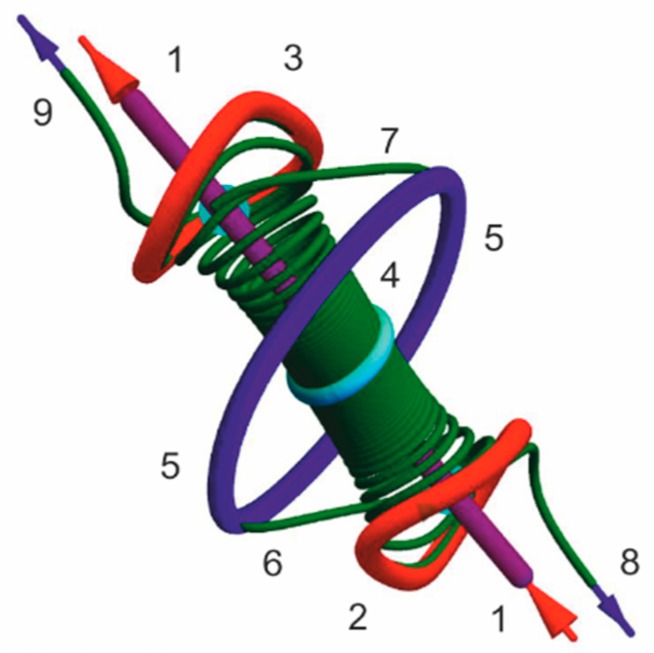
Energy flows for the “precesson”. The only vortex line 1 is oriented according to red arrows (m=1). It includes three special points where tangential component of energy flow changes sign. Around the special points there are closed lines of energy flow 2, 3, 4 (2 and 3 are unstable and 4 is saddle limit cycles). Vortex tube is a boundary surface of domain of attraction of energy flow lines in neighborhood of vortex lines 1. It is formed by separatrix energy flow lines beginning on unstable limit cycles 2 or 3 and ending on stable limit cycle 5 (like trajectories 6 and 7) or going to the periphery (like 8 and 9).

In Figure 13 we describe such field for the “precesson”. The only vortex line 1 is alternating: it includes three special points of change of direction of tangential component of energy flow for neighboring lines. The special point is situated inside closed lines of energy flow that are unstable (a line of flow moves away from these points when its length *l* increases). Near the vortex line at the periphery, energy flows in opposite directions—from the centre to the periphery. The vortex line attracts the lines of energy flow inside a vortex tube bounded by a surface formed by separatrix flow lines starting on stable limit cycle 5 and ending on stable limit cycle 5 or going to the periphery. 

**Figure 14:**
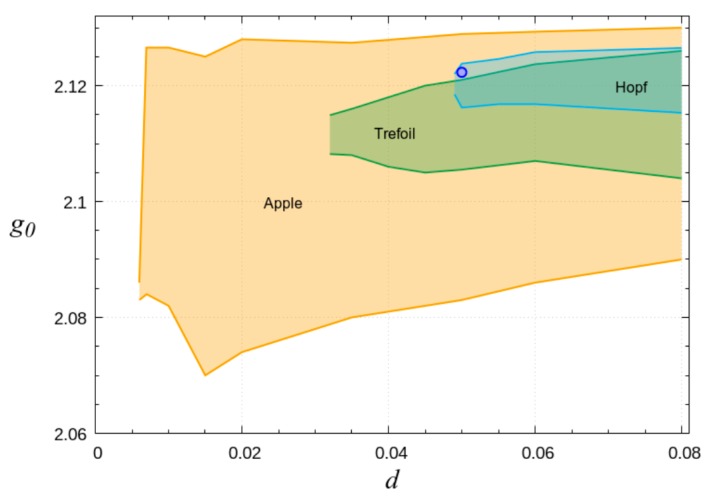
Domains of stability of three types of tangle laser solitons: “apple”, “Hopf+”, and “trefoil+”. The domains overlap; those for “Hopf+” and “trefoil+” are inside the widest domain of “apple” solitons.

Different types of topological laser solitons have different domains of stability in the parameter space. The widest domain is for the “apple” soliton (Figure 14). The stability domains of more complex topologically solitons are shifted towards larger values of effective diffusion coefficient *d*. Important is overlapping of stability domains for different types of solitons—“apple”, “Hopf+”, and “trefoil+” (here sign “+” denotes that skeleton is formed from closed vortex lines like Hopf link or trefoil and from unclosed vortex lines). It means that for the same scheme parameters, it is possible to generate topologically different solitons properly preparing their initial structure.

### 3.3. Hysteresis

Here, we investigate the effect of a change in the scheme parameters on the characteristics of topological laser solitons. As we have seen, topology does not uniquely determine the type of soliton. Thus, the “apple” solitons coexisting in certain ranges of parameters have the same topology. Differences appear in the degree of asymmetry and in the values of a number of integral characteristics. These characteristics include electromagnetic energy of soliton

(40) $$W(z)=\int |E(r_{3},z){|}^{2}dr_{3},$$

torque

(41) M(z)=∫r3×Im(E*∇rE)dr3,

and inertia tensor $\hat{J}(z)$ with matrix elements

(42) $$J_{ij}(z)=\int (r_{i}^{2}\delta_{ij}-r_{i}r_{j})|E(r_{3},z){|}^{2}dr_{3},$$

where $\delta_{ij}$ is the Kronecker symbol. **M** and $\hat{J}$ are calculated with respect to the structure center

(43) $$R_{c}(z)=\int r_{3}|E(r_{3},z){|}^{2}dr_{3}/W(z).$$

Eigenvectors of inertia tensor, i.e., three mutually orthogonal principal axes of tensor $\hat{J}$, form a trihendron characterizing the intensity distribution orientation. After determination of such orientation, one can introduce a *z*-dependent vector of angular velocity Ω. Of interest is also not the energy of the medium itself (in the model of medium with infinite size, it is infinite), but its deviation from the equilibrium value in the absence of radiation

(44) $$\delta W_{m}(z)=\int [f(I(r_{3},z)-f(0)]\;dr_{3}.$$

**Figure 15:**
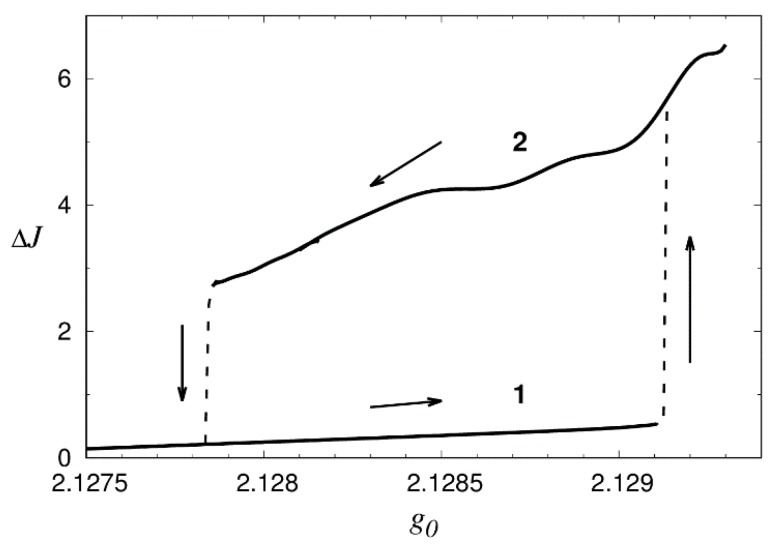
Hysteretic dependence of difference of two main inertia moments of “apple” laser solitons with the same topology. In state 1 soliton has fixed “solid-like” structure, whereas it is more asymmetric and oscillates in state 2.

The “apple” solitons are most sensitive to such characteristics as the main inertia moments (eigenvalues of inertia tensor), and more exactly to the difference of two moments close in modulus ΔJ, Figure 15. With slow increase of small-signal gain $g_{0}$ soliton keeps stable “solid-like” structure up to the value $g_{0}=2.1291$. Then, it switches rapidly to more asymmetric and oscillating state 2. With further decrease of $g_{0}$, soliton switches off to state 1 at $g_{0}=2.1278$. Thus, there is a classical loop of reversible hysteresis.

**Figure 16:**
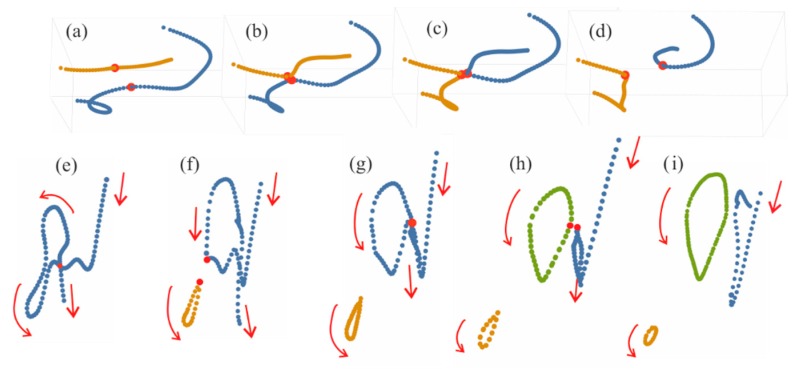
Two types of topological reactions with vortex lines: their reconnection (**a**–**d**) and separation of loops from the parent line (**e**–**i**). Evolution coordinate *z* increases from (**a**) to (**d**) and from (**e**) to (**i**).

More complicated is the dynamics of hysteresis when topological structure of solitons changes during hysteresis cycle. The skeleton transformations include topological reactions with vortex lines of the two types. First, two vortex lines can reconnect with exchange of their branches (Figure 16, upper row). Second, closed vortex loops with unit topological charge can separate from the parent vortex line after its sharp bending (Figure 16, lower row). Note that similar elementary transformations can also occur in systems without energy sources [66,67,68,69,70,71,72]. However, it is difficult to talk about hysteresis phenomena in such systems without stable attractors.

**Figure 17:**
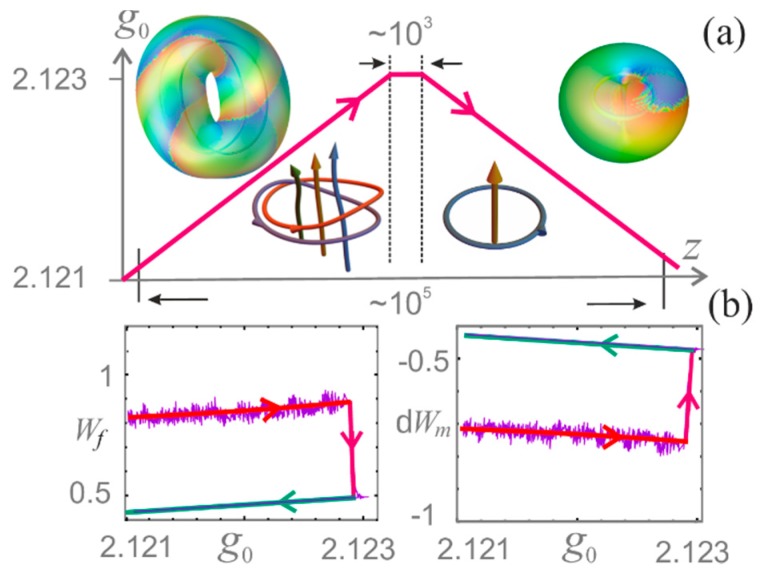
(**a**) Cycle of variation of small-signal gain $g_{0}$ (red trapezoidal line). Inserts: isointensity surfaces and skeletons for “Hopf+” (left, increase of $g_{0}$) and “apple” (right, decrease of $g_{0}$) solitons. (**b**) Variation of the field energy $W_{f}$ (left) and energy of the medium $dW_{m}$ (right) during increase (red) and decrease (green) of $g_{0}$.

We now indicate the main features of the hysteresis for the initial soliton “Hopf+”. The slow variation of small-signal gain $g_{0}$ is shown in Figure 17a. At the stage of increasing $g_{0}$, this soliton retains its “solid-state” behavior, but loses stability when crossing the boundary of the region indicated in Figure 14. Here at the stage of stabilization of $g_{0}$, after numerous topological reactions, the “Hopf+” soliton turns into an “apple” soliton, retaining this type with a further decrease in $g_{0}$. When $g_{0}$ restores the original value, this does not happen with the soliton topology. It is simplified (reduction of topological indices). In addition, the electromagnetic energy of the soliton decreases, and the energy of the medium increases (Figure 17b). Thus, under the conditions of a change in the topological structure of the soliton, the hysteresis is irreversible. 

## 4. Conclusions

As was shown above, nanostructuring of media in the form of molecular J-aggregates or nanosized organic film allows one to form extremely narrow spatial structures. As for generation of extremely short laser pulses, promising are such nanostructures as matrices with imbedded quantum dots [22,73].

Another face of structuring is the direct shaping of light packets, ultimately in 3D-variant. The presented “Hula-hoop” or tangle laser solitons are based on resonance response of media with amplification and absorption and have therefore lower energetic threshold of generation. Further decrease of the threshold is possible with nanostructured optical media with higher dipole moments of resonance transitions. In this case, the use of topological 3D-laser solitons would allow developing a new approach to reliable processing of optical information.
